# Yogurt and molasses can alter microbial-digestive and nutritional characteristics of pomegranate leaves silage

**DOI:** 10.1186/s13568-022-01452-4

**Published:** 2022-09-01

**Authors:** Mohsen Kazemi, Reza Valizadeh, Elias Ibrahimi Khoram Abadi

**Affiliations:** 1 Department of Animal Science, Faculty of Agriculture and Animal Science, University of Torbat-e Jam, Torbat-e Jam, Iran; 2 Department of Animal Science, Faculty of Agriculture, Ferdowsi University of Mashhad, Mashhad, Iran

**Keywords:** Pomegranate leaves, Silage, Yogurt, Additive, Nutritional value

## Abstract

Fewer studies in recent years have been conducted on the nutritional potential and fermentation quality of silage prepared from pomegranate leaves (PL). So, we investigated the nutritional-fermentation quality of PL before and after ensiling with or without yogurt containing mainly lactic acid-producing bacteria (*Lactobacillus bulgaricus* and *Streptococcus thermophiles*) and molasses (at two levels of 2 and 4% of dry matter) in the polyethylene microsilos for 60 days. A range of dry matter (29.1–39.1%), crude protein (3.85–4.83%), ash (5.33–8.60%), and non-fiber carbohydrates (53.2%–58.6%) contents were observed among the treatments. A significant increase in calcium, potassium, magnesium, manganese, iron, and zinc was observed in PL after ensiling compared to before ensiling (*p* < 0.05). The PL ensiled with 4% yogurt exhibited the highest ammonia nitrogen, lactic and acetic acids, but the lowest butyric acid among the ensiled PL (*p* < 0.05). The ensiling of PL without additive (control) significantly decreased potential gas production, dry matter digestibility, organic matter digestibility, total volatile fatty acids, metabolizable energy, net energy for lactation, base-buffering capacity, titratable alkalinity, and acid–base buffering capacity compared to before ensiling (*p* < 0.05). According to the present results, the nutritional value of PL before ensiling was higher than after ensiling. The addition of yogurt and molasses to PL at the ensiling process especially at 4% of dry matter, improved the fermentation and nutritional characteristics. In general, the addition of yogurt or molasses as two cheap and available additives is recommended to improve the digestive-fermentation parameters of PL in silo and ruminal environments.

## Introduction

As a result of population growth and recent droughts, the use of unconventional foods due to their low-cost and good nutritional value has attracted the attention of ruminant husbandries. Unconventional feedstuffs are those that are less commonly used to balance the diet of ruminants, and little research has been done on their nutritional properties and nutritional value. Pomegranate (*Punica granatum* L.) trees are cultivated in arid, semi-arid, and Mediterranean areas and with high extensive in Iran (Taher-Maddah et al. 2012). In a study, the chemical-mineral composition and nutritional potential of PL have been investigated by Kazemi and Mokhtarpour (2021). Ensiling is a common way to store forage with high moisture content. The primary purpose of ensiling is to ensure the conversion of water-soluble carbohydrates to organic acids by mainly lactic acid-producing bacteria. Different additives are added to silage to improve the fermentation quality of prepared silages by producing more lactic acid (McDonald 1981). Although different additives are used when ensiling for different purposes, the main goal is to prevent secondary fermentation and reduce butyric acid production (Aksu et al. 2004). Feeding ruminants with favorite silage can improve the quality of the diet and ultimately increase their performance. Inhibition of the growth of undesirable bacteria in the silage environment is related to the rate of lactic acid production, which depends on the initial population of lactic acid bacteria and the availability of the substrate (McDonald et al. 1991). Yogurt is an inexpensive and available additive that contains natural lactic acid-producing bacteria. It is also a fermented dairy product produced by lactic acid bacteria such as *Streptococcus thermophilus* and *Lactobacillus delbrueckii* subsp. *bulgaricus* (Nagaoka 2019). During yogurt preparation, these bacteria produce lactic acid, decreasing pH and causing milk protein to coagulate (Nagaoka 2019). Silage additives such as molasses and bacterial inoculants have been used to reduce the pH of the silo environment (Baytok et al. 2005; Li et al. 2019). Recent studies have shown the benefits of pomegranate waste (peels, seed, pith, carpellary membrane, leaves and arils) as an unconventional feedstuff in ruminant feeding (Natalello et al. 2019; Kazemi and Mokhtarpour 2021; Kazemi and Valizadeh 2021). There was no data about the silage prepared from PL, so we investigated chemical-mineral composition, fermentation-digestive characteristics, and buffering capacity of PL before and after ensiling with two additives (yogurt and molasses), each one at two levels of 2 and 4% of dry matter (DM). We hypothesized that molasses and yogurt addition could improve the fermentation-digestive characteristics and the nutritional quality of PL after ensiling.

## Materials and methods

### Silage preparation

The samples of pomegranate leaves (PL) from different trees were collected in autumn 2020 from four gardens located in Kashmar (35˚2434′N latitude and 58˚4687′E longitude), Iran. The mean annual rainfall of Kashmar is 206 mm with a mean annual temperature of 17.7 °C and an arid and semi-arid climate in autumn (Kazemi and Mokhtarpour 2021). Some of the collected leaves were wholly mixed and immediately transferred to the laboratory, dried in an oven (Behdad Co.) at 60 °C for 48 h, ground through a 1-mm mesh screen in a Wiley Mill, and preserved in polyethylene bags for the subsequent analysis. Other samples with or without additives were ensiled in polyethylene containers with a capacity of 1.5 kg. Experimental treatments included (1) fresh PL before ensiling, (2) fresh PL after ensiling [without additive, control], (3) fresh PL ensiled with 2% yogurt (DM basis), (4) fresh PL ensiled with 4% yogurt (DM basis), (5) fresh PL ensiled with 2% molasses (DM basis), (6) fresh PL ensiled with 4% molasses (DM basis). Each additive was added to PL at the appropriate level (2 or 4% of DM), then PL was moved to microsilos, compacted, and ensiled for 60 days. Four replications were considered for each treatment. Molasses was prepared from the Torbat-e Jam sugar factory, Iran. A commercial yogurt was also prepared from a factory located in Torbat-e Jam, Iran. The tested yogurt mainly contained 1.2 × 10^9^ colony forming units (CFU)/gram.

### Chemical-mineral analysis and fermentation end-product of silage

After 60 days of ensiling, each silo was separately opened and emptied, and its contents were sampled. A fresh sample of each treatment was also dried in an air-forced oven at 135 °C for 4 h (AOAC 2005) for DM determination. To determine the pH of silage, 450 ml of distilled water was added to 50 g of the sample, mixed with an electric mixer for five minutes, filtered via four layers of cheesecloth, and finally, the pH of the extract was determined by a pH meter (Eyni and Bashtani 2016). A sub-sample of silage extract was employed for lactic, acetic, propionic, and butyric acids analysis by a KNAUER HPLC system equipped with a UV–VIS detector (Azura, Germany) and with a C18 columns (25 cm × 4.6 mm id, 5 μm). A mobile phase of 0.005 mM H_2_SO_4_ with a flow rate of 0.5 mL/min was considered for the operation. To measure ammonia nitrogen, 10 ml of silage extract was mixed with 10 ml of 0.2 N HCl and kept in the freezer at -18 °C until subsequent analysis. The neutral detergent fiber (NDF) and acid detergent fiber (ADF) were determined according to the procedure of Ankom technology (Ankom Technology 2006a, b) using solutions described by Van Soest et al. (1991). The Kjeldahl method (AOAC 2005) was employed for crude protein (CP) determination. The ether extract (EE) determined by Soxhlet extracting apparatus (AOAC 2005). The content of non-fiber carbohydrates (NFC) in samples was calculated by subtracting CP, NDF, EE, and ash from total DM (Sniffen et al. 1992). The ash content was determined using a furnace with a temperature of 550 °C for 4 h. The concentration of phosphorus was determined by a spectrophotometer (UV–Vis array Spectrophotometer, Photonix-Ar-2017, Iran) using the molybdovanadate method. The mineral contents of samples, including calcium, sodium, potassium, magnesium, manganese, iron, and zinc were determined by atomic absorption spectrometry (SavantAA, GBC, Australia).

### In vitro protocol

We used the method described by Menke and Steingass (1988) for a gas test run. Two runs were considered for the gas test procedure. Three ruminal fistulated lambs were employed for rumen fluid collection. They fed on alfalfa hay and a commercial concentrate twice (7:0 am and 18:0 pm) a day at the maintenance level. The rumen fluid was strained via four layers of cheesecloth, flushed with CO_2_, transferred into a pre-warmed thermos flask, and then transferred to the laboratory for the subsequent analysis. The amount of 200 mg of each sample was weighed into the 100 ml glass syringes. The artificial saliva was prepared according to Menke and Steingass (1988) procedures. Each glass syringe was filled with rumen fluid and artificial saliva solution with a ratio of 1:2. The syringe outlet was closed by a plastic clip. Afterward, each syringe was then gently shaken and placed in a water bath at 39 °C. The gas volume was recorded continuously at 3, 6, 9, 12, 24, 48, 72, and 96 h of incubation (Menke and Steingass 1988). The 24 h gas production, CP, and EE were employed to estimate the metabolizable energy (ME) and net energy for lactation (NEl) according to equations described by Menke and Steingass (1988). A medium similar to the gas test was used to determine total volatile fatty acids (TVFA), pH, and ammonia nitrogen concentrations. The sampling for TVFA determination was conducted according to methods described by Getachew et al. (2004). The Markham device (Markham 1942), based on steam distillation, was used for TVFA determination according to the protocol described by Barnett and Reid (1957). The 24 h organic matter and dry matter digestibility (OMD and DMD) of samples was conducted according to Kazemi and Ghasemi Bezdi (2021) procedure. The method of Komolong et al. (2001) was used for ammonia nitrogen determination. The buffering capacity parameters were determined according to Jasaitis et al. (1987).

### Statistical analysis and equations

For each measured parameter, four replications were considered. All data were analyzed in a completely randomized design using the GLM procedure of SAS (2002). The statistical differences between means of treatments were determined by Duncan’s multiple range test (Kazemi et al. 2012). The parameters related to the gas production, including fractional rate (c_gas_, %/h) and potential gas production (b_gas_, ml/h), were determined by a nonlinear equation [Ørskov and McDonald 1979; $Y=b\left(1-{e}^{-ct}\right)$], where Y is the volume of gas produced at time t.

## Results

### Chemical-mineral concentrations

Chemical compositions of fresh PL or ensiled with different additives are presented in Table 1. We observed a different range of chemical composition among the treatments. Compared to the PL ensiled without additive, silages containing 4% molasses or 4% yogurt exhibited the highest DM content (*p* < 0.0001). The concentration of EE was unchanged among the ensiled and non-ensiled PL (*p* > 0.05), while the lowest ash content was observed in PL ensiled with 4% molasses (5.33%, *p* = 0.013). PL after ensiling (without additive, control) had the lowest NFC compared to the other treatments (53.2%, *p* = 0.002). Silages containing additives had lower NDF (*p* = 0.05) and ADF (*p* = 0.004) compared to the control silage.

**Table 1:** Chemical compositions (% of DM) of fresh pomegranate leaves or ensiled with different additives

Treatment	DM	NDF	ADF	CP	EE	Ash	NFC
PL before ensiling	38.2^a^	27.8^ab^	19.7^ab^	4.12^c^	5.36	7.37^a^	56.8^ab^
PL after ensiling	29.1^c^	29.5^a^	21.7^a^	4.83^a^	4.85	8.60^a^	53.2^c^
PL ensiled 2% yogurt	32.8^b^	27.3^b^	19.5^b^	4.42^b^	5.01	8.33^a^	54.9^bc^
PL ensiled 4% yogurt	38.2^a^	27.0^b^	18.4^b^	3.90^d^	4.78	7.28^a^	57.1^ab^
PL ensiled 2% molasses	33.4^b^	27.6^b^	19.2^b^	4.27^bc^	4.80	6.92^ab^	56.3^ab^
PL ensiled 4% molasses	39.1^a^	27.2^b^	18.8^b^	3.85^d^	5.00	5.33^b^	58.6^a^
SEM	0.80	0.57	0.45	0.05	0.24	0.54	0.69
P-value	< 0.0001	0.05	0.004	< 0.0001	0.58	0.013	0.002

Means within columns followed by the same letter are not different

*PL* pomegranate leaves, *DM (% of fresh weight)* dry matter, *NDF* neutral detergent fiber, *ADF* acid detergent fiber, *CP* crude protein, *EE* ether extract, *NFC* non-fiber carbohydrate

*SEM* standard error of the mean

Mineral compositions of fresh PL or ensiled with different additives are presented in Table 2. We also observed a different range of mineral composition among the treatments. Exception sodium (p = 0.34), we found an increasing effect for other minerals including calcium (*p* = 0.0001), phosphorus (*p* = 0.05), potassium (*p* = 0.07), magnesium (*p* = 0.005), manganese (*p* = 0.0009), iron (*p* < 0.0001), and zinc (*p* = 0.0004) in control silage rather than before ensiling. Addition each of additives (yogurt and molasses) to PL, significantly decreased the concentrations of calcium (*p* = 0.0001), phosphorus (*p* = 0.05), potassium (*p* = 0.07), magnesium (*p* = 0.005), manganese (*p* = 0.0009), iron (*p* < 0.0001), and zinc (*p* = 0.0004) compared with the control silage.

**Table 2:** Mineral compositions of fresh pomegranate leaves or ensiled with different additives

Treatment	Ca	P	Na	K	Mg	Mn	Fe	Zn
PL before ensiling	19.7^bc^	1.70^b^	0.53	2.67^c^	2.60^d^	25.5^b^	155^c^	6.33^d^
PL after ensiling	29.8^a^	2.57^a^	0.78	5.24^a^	4.11^a^	36.8^a^	438^a^	12.9^a^
PL ensiled 2% yogurt	21.4^b^	1.82^b^	0.82	3.62^bc^	3.56^abc^	31.5^a^	340^b^	10.0^bc^
PL ensiled 4% yogurt	15.4^c^	1.73^b^	0.78	4.25^ab^	3.08^bcd^	23.9^b^	302^b^	9.57^bc^
PL ensiled 2% molasses	21.5^b^	2.07^ab^	0.71	3.56^bc^	3.67^ab^	31.3^a^	372^ab^	11.5^ab^
PL ensiled 4% molasses	16.5^c^	1.80^b^	0.53	3.68^bc^	2.83^cd^	23.3^b^	303^b^	8.49^c^
SEM	1.36	0.19	0.12	0.36	0.23	1.78	22.5	0.70
P-value	0.0001	0.05	0.34	0.007	0.005	0.0009	< 0.0001	0.0004

Means within columns followed by the same letter are not different

*PL* pomegranate leaves, *Ca (g/kg DM)* calcium, *P (g/kg DM)* phosphorus, *Na (g/kg DM)* sodium, *K (g/kg DM)* potassium, *Mg (g/kg DM)* magnesium (g/kg DM), *Mn (mg/kg DM)* manganese, *Fe (mg/kg DM)* iron, *Zn (mg/kg DM)* zinc

*SEM* standard error of the mean

### Fermentation quality of silage prepared from pomegranate leaves

The fermentation quality of PL ensiled with different additives is presented in Table 3. The addition of 2 and 4% yogurt or molasses significantly decreased the pH of the silage environment compared to the control silage (*p* = 0.02). The propionic acid concentration remained unchanged among the prepared silages (*p* > 0.05). Compared to the control silage, the highest concentration of lactic (*p* = 0.0007) and acetic (*p* = 0.02) acids and ammonia nitrogen (*p* < 0.0001) and lowest concentration of butyric acid (*P* = 0.0003) were observed in PL ensiled with 4% yogurt. Also, the addition of molasses and yogurt increased the concentrations of lactic and acetic acids and decreased butyric acid concentration compared to the control silage.

**Table 3:** Fermentation quality of pomegranate leaves ensiled with different additives

Treatment	LA	AA	PA	BA	AN	pH
PL before ensiling	–	–	–	–	–	–
PL after ensiling	2.55^c^	0.93^c^	0.74	0.66^a^	0.31^e^	4.32^a^
PL ensiled 2% yogurt	3.01^b^	1.33^ab^	0.81	0.33^bc^	0.73^b^	3.98^b^
PL ensiled 4% yogurt	3.60^a^	1.62^a^	0.83	0.22^c^	1.13^a^	4.06^b^
PL ensiled 2% molasses	3.08^b^	1.12^bc^	0.80	0.40^b^	0.44^d^	4.03^b^
PL ensiled 4% molasses	3.30^ab^	1.47^ab^	0.84	0.33^bc^	0.61^c^	4.00^b^
SEM	0.11	0.12	0.03	0.04	0.03	0.06
P-value	0.0007	0.02	0.33	0.0003	< 0.0001	0.02

Means within columns followed by the same letter are not different

*PL* pomegranate leaves, *LA (% of DM)* lactic acid, *AA (% of DM)* acetic acid, *PA (% of DM)* propionic acid, *BA (% of DM)* butyric acid, *AN (% of total nitrogen)* ammonia nitrogen

*SEM* standard error of the mean

### Gas production and ruminal fermentation parameters

Some ruminal fermentation parameters measured in the culture medium after incubation of fresh PL or ensiled with different additives are presented in Table 4. Ensiling of PL without additive (control) decreased b_gas_ (*p* < 0.0001) and 12 (*p* = 0.0004), 24 (*p* < 0.0001), 48 (*p* < 0.0001), 72 (*p* = 0.0002) h gas production compared to before ensiling. Also, addition of molasses or yogurt (2 and 4% of DM) to PL silage significantly increased 12 (*p* = 0.0004), 24 (*p* < 0.0001), 48 (*p* < 0.0001), 72 (*p* = 0.0002) h cumulative gas production compared to the control silage.

**Table 4:** The gas production parameters of fresh pomegranate leaves or ensiled with different additives

Treatment	b_gas_	c_gas_	gas 12 h	gas 24 h	gas 48 h	gas 72 h
PL before ensiling	35.0^a^	0.026^c^	6.57^ab^	18.9^a^	26.2^a^	28.8^a^
PL after ensiling	19.2^b^	0.024^c^	2.40^c^	10.6^c^	14.6^c^	15.5^c^
PL ensiled 2% yogurt	24.1^b^	0.031^b^	5.33^b^	14.7^b^	19.6^b^	20.7^b^
PL ensiled 4% yogurt	32.8^a^	0.033^ab^	8.40^a^	21.6^a^	27.9^a^	28.0^a^
PL ensiled 2% molasses	23.0^b^	0.032^ab^	5.50^b^	14.3^b^	18.9^b^	20.1^bc^
PL ensiled 4% molasses	30.5^a^	0.036^a^	8.43^a^	20.0^a^	26.2^a^	27.6^a^
SEM	1.62	0.0015	0.69	1.07	1.35	1.51
P-value	< 0.0001	0.0008	0.0004	< 0.0001	< 0.0001	0.0002

Means within columns followed by the same letter are not different

*PL* pomegranate leaves, *b*_*gas*_* (ml/200 mg DM)* potential gas production, *c*_*gas*_* (%/h)* fractional rate of gas production; gas 12, 24, 48, and 72 h (ml/200 mg DM): cumulative gas production after 12, 24, 48, and 72 h incubation

*SEM* standard error of the mean

Some ruminal fermentation parameters measured in the culture medium after incubation of fresh PL or ensiled with different additives are shown in Table 5. The pH in the culture medium was not affected by the experimental treatments (*p* > 0.05). We found a significantly higher TVFA (*p* < 0.0001), OMD (*p* < 0.0001), DMD (*p* < 0.0001), ME (*p* = 0.0004), NEl (*p* = 0.0003), and a significantly lower ammonia nitrogen (*p* = 0.0006) in PL before ensiling compared to the control silage. Addition of two additives (2 and 4% DM) significantly decreased ammonia nitrogen in the culture medium compared to the control silage (*p* = 0.0006). Also, the amounts of TVFA (*p* < 0.0001), OMD (*p* < 0.0001), DMD (*p* < 0.0001), ME (*p* = 0.0004), and NEl (*p* = 0.0003) were significantly higher in the silages containing 2 and 4% yogurt or molasses compared to the control silage.

**Table 5:** Some ruminal fermentation parameters measured in the culture medium after incubation of fresh pomegranate leaves or ensiled with different additives

Treatment	NH_3_-N	TVFA	pH 24 h	OMD	ME	NEl	DMD
PL before ensiling	15.5^cd^	63.4^a^	6.81	72.8^a^	5.82^a^	3.01^abc^	69.1^a^
PL after ensiling	16.3^a^	59.8^c^	6.90	51.0^d^	4.58^c^	2.15^d^	46.7^d^
PL ensiled 2% yogurt	15.9^b^	61.6^b^	6.92	57.7^c^	5.17^b^	2.56^bcd^	53.7^c^
PL ensiled 4% yogurt	15.3^d^	64.6^a^	6.91	72.8^a^	6.01^a^	3.15^a^	68.9^a^
PL ensiled 2% molasses	15.9^b^	61.5^b^	6.87	58.1^c^	5.05^bc^	2.48^cd^	54.2^c^
PL ensiled 4% molasses	15.8^bc^	63.9^*a*^	6.84	64.6^b^	5.85^a^	3.04^ab^	60.7^b^
SEM	0.12	0.46	0.08	0.41	0.17	0.11	0.57
P-value	0.0006	< 0.0001	0.91	< 0.0001	0.0004	0.0003	< 0.0001

Means within columns followed by the same letter are not different

OMD and DMD were determined after 24 h incubation

*PL* pomegranate leaves, *NH*_*3*_*-N (mg/dL)*: ammonia nitrogen, *TVFA (mmol/L)* total volatile fatty acid, *OMD (%)* organic matter digestibility, *ME (MJ/kg DM)* metabolizable energy, *NEl (MJ/kg DM)* net energy for lactation, *DMD (%)* dry matter digestibility

*SEM* standard error of the mean

### Buffering capacity

The pH of the extract and buffering capacity (mEq × 10^–3^) parameters of pomegranate leaves ensiled with different additives are exhibited in Table 6. Among the treatments, the highest amounts of pH and titratable acidity were observed in the control silage (*p* < 0.0001). Also, the highest amounts of acid-buffering capacity (110 mEq × 10^–3^) and acid–base buffering capacity (194 mEq × 10^–3^) were related to PL ensiled with 4% molasses (*p* < 0.0001). The amounts of titratable alkalinity (436 mEq × 10^–3^) and base-buffering capacity (86.1 mEq × 10^–3^) were highest in PL ensiled with 2% yogurt (*p* < 0.0001).

**Table 6:** The pH of the extract and buffering capacity (mEq × 10^–3^) parameters of fresh pomegranate leaves or ensiled with different additives

Treatment	pH	Titratable acidity	Acid-buffering capacity	Titratable alkalinity	Base-buffering capacity	Acid–base buffering capacity
PL before ensiling	4.18^b^	14.0^b^	77.4^b^	368^c^	77.5^c^	155^b^
PL after ensiling	4.27^a^	21.7^a^	80.2^b^	245^d^	51.5^d^	132^c^
PL ensiled 2% yogurt	3.92^d^	0.00^d^	0.00^c^	436^a^	86.1^a^	86.1^d^
PL ensiled 4% yogurt	3.89^d^	0.00^d^	0.00^c^	410^b^	81.4^b^	81.4^d^
PL ensiled 2% molasses	3.95^d^	0.00^d^	0.00^c^	422^ab^	83.4^ab^	83.4^d^
PL ensiled 4% molasses	4.10^c^	11.0^c^	110^*a*^	413^b^	84.3^ab^	194^a^
SEM	0.02	0.43	4.21	5.86	1.04	4.05
P-value	< 0.0001	< 0.0001	< 0.0001	< 0.0001	< 0.0001	< 0.0001

Means within columns followed by the same letter are not different

*PL* pomegranate leaves

*SEM* standard error of the mean

## Discussion

### Chemical-mineral concentrations

The DM (38.2 vs. 38.4%), NDF (27.8 vs. 26.4%), and ADF (19.7 vs. 20.1%) concentrations of PL before ensiling were close to those reported by Kazemi and Mokhtarpour (2021). The primary purpose of silage preparation with higher quality is to minimize DM losses and maintain the maximum aerobic stability and nutritive value using different additives or new methods. Some of the quality of silages can be evaluated by chemical analyses. The treatment of the present silages with or without additives compared to before ensiling affected some chemical composition (DM, CP, Ash, and NFC) after 60-day ensiling. The range of DM of the prepared silages was between 29.1% and 39.1%, which was within an ideal range reported by Ergün et al. (2001) for different silages. In line with other reports (Li et al. 2014, 2019; Wang et al. 2019), we found an increase in DM content of PL ensiled with additives compared to the control silage. This increase can be attributed to the growth of lactic acid-producing bacteria, which reduces the pH of the silo environment, then inhibits the growth of harmful anaerobic bacteria, reduces the consumption of nutrients by these microorganisms, then preserves nutrients, and finally result in the increase of DM content of silages containing additives (yogurt and molasses). In line with the present study, Baytok et al. (2005) reported an increase in DM content of corn silage when molasses was added to the silages. To make good quality silage, it is necessary to produce silage from wilted material that contains DM between 32 to 38% (Knotek 1997). Also, it is reported that the DM content of the primary substrate for ensiling should be above 35% to ensure successful fermentation (Đorđević et al. 2001). Therefore, according to the above reports, PL had an ideal DM content (about 38.2%) for ensiling. An increase in CP content of PL after ensiling compared to before ensiling could be attributed to the concentration effect due to the loss of organic carbon during fermentation or the combination of proteolysis inhibition and concentration effect (He et al. 2018). Also, the increase in CP content of the control silage compared to before ensiling was not only associated with increased protein supply but was also associated with a stable rate of proteolysis during ensiling, as evidenced by close percentages of DM loss due to ensiling. However, the primary mechanism underlying such findings remained ambiguous. We found that the PL ensiled with additives had lower ADF, EE, and ADF contents compared with the PL before ensiling. Consistently, other studies have reported that molasses or other additives can decrease the fiber fractions (NDF and ADF) of silages (Li et al. 2014; Babaeinasab et al. 2015).

We found an increase of minerals (exception sodium) in the control silage compared to PL before ensiling and also a decrease in minerals content (calcium, phosphorus, potassium, magnesium, manganese, iron, and zinc) between silages containing additives or no additives. The decrease in the amounts of minerals after ensiling of PL can be attributed to the use of organic matters by microorganisms, which has ultimately led to an increase in minerals.

### Fermentation quality of silage

The rate of pH reduction is considered as an essential indicator for reflecting microbial activity and silage fermentation (Ni et al. 2017). In this study, all silages containing additives showed lower pH values than the control silage. In general, lactic acid because of its stronger acid characteristics (pK_a_ 3.86) compared to acetic (pK_a_ 4.76) is the final goal of the end product of fermentation in the silage (Muck 2010). High concentrations of lactic acid lead to a rapid decrease in the pH of silage, which reduces the activity of harmful microorganisms and the production of butyric acid. A decrease in pH of ensiled PL with additives can be related to their higher lactic and acetic acids production compared to the control silage. The pH value (3.98–4.06, Table 3) of the ensiled PL with additives suggests that these silages underwent a proper fermentation process. Some researchers suggested a pH range of 3.8–4.2 as indicative of good fermentation for silage of tropical grass (Rabelo et al. 2019). Kleinschmit and Kung (2006) reported that microbial inoculants decreased the pH of the silo environment. A higher concentration of ammonia nitrogen in the ensiled PL with additives can be attributed to excessive protein breakdown caused by a slow drop in pH (Gandra et al. 2016). Higher butyric acid in the control silage can be related to yeast activity (Gandra et al. 2016). As we found a high level of butyric acid (0.66% of DM) in the control silage, Kung and Shaver (2001) reported that a high level of butyric acid (> 0.5% of DM) indicates the clostridial fermentation, which is one of the unsuitable fermentations. It is also reported that silages containing high levels of butyric acid are usually low in nutritive value and have higher ADF and NDF levels because many of the soluble nutrients have been degraded. In this regards, we also found that control silage had high level of butyric acid as well as higher NDF and ADF rather than other prepared silages. In line with our results, the addition of whey as a natural source of bacteria improved the fermentation characteristics of alfalfa silage (Mariotti et al. 2020). The present additives increased lactic acid and acetic acid production as well as ammonia nitrogen in the silo environment simultaneously, which its reason is unknown. This simultaneous increase may be due to the different nature of pomegranate leaves. It is reported that if the concentration of ammonia nitrogen is less than 10% of total nitrogen, fermentation quality is ideal, as in our study, the concentration of ammonia nitrogen was less than this value (McDonald et al. 2010). Although two applied additives (yogurt and molasses) increased ammonia nitrogen in silages, the amount of ammonia nitrogen in these silages was normal (below 10% of total nitrogen). When the pH value is high enough to limit the activity of proteolytic bacteria, the proteins in the fresh material are preserved (Man and Wiktorsson 2002). So, although the different CP contents of the PL silages in our study cannot prove the efficiency of additives in protein preservation, however, the proteolytic activity of microorganisms inside the PL silages seems to be negligible. In line with the present study, Steg and Meer (1985) reported that the addition of molasses to silage increased acetic and lactic acids compared to the control silage. They suggested that the use of molasses stimulated the growth of hetero-fermentative instead of homo-fermentative lactic acid bacteria due to the specific source of water-soluble carbohydrates in molasses, i.e., sucrose (Steg and Meer 1985). Similar to our results, the addition of molasses to corn silage increased lactic acid and ammonia nitrogen compared to the control group (without additive) (Baytok et al. 2005).

### Gas production and ruminal fermentation parameters

Due to its ease of implementation and low cost, the gas production technique can easily be used to evaluate feedstuffs and forages. Gas production reflects the produced short-chain fatty acids in gas production techniques and provides essential information on the ruminal fermentation of phytochemicals and animal feed (Makkar 2005). We found PL before ensiling or ensiled PL with two additives had higher b_gas_ and 12, 24, 48, and 72 h gas production rather than the control silage. The increased gas production can be attributed to their higher NFC content rather than the control silage. When a feedstuff or forage is incubated in vitro, the carbohydrates are fermented to the short-chain fatty acids, gases (mainly CO_2_ and CH_4_), and microbial cells (Getachew et al. 1998). In the present study, higher (12.5%) potential gas production (35 vs. 30.6 ml/200 mg DM) for PL before ensiling was observed than that reported by Kazemi and Mokhtarpour (2021).

A strong correlation between DMD, OMD, and b_gas_ with TVFA was reported by Kazemi and Valizadeh (2019) and Kazemi (2019). So, higher TVFA in PL before ensiling or ensiled PL with 4% yogurt or molasses can be attributed to more DMD, OMD, and b_gas_. One of the most critical applications of the gas production technique is determining the digestibility and energy value of animal feed (Krishnamoorthy et al. 2005). We found PL before ensiling or PL ensiled with two additives had higher metabolism energy rather than the control silage. This increase can be attributed to their higher 24 h gas production. In line with our results, the addition of 1% of a commercial yogurt containing *Lactobacillus plantarum*, *L. bulgaricus*, *L. casei*, *L. acidophilus*, and *L. bifidus* as a natural source of bacteria increased the in vitro DMD and OMD of sugarcane silage (Reyes-Gutiérrez et al. 2018). One of the most critical be attributed to higher content indicators of feed evaluation is determining its digestibility, which can affect feed intake and largely relies on chemical compounds, especially the fibrous and structural parts of the plant (Chabot et al. 2008). So, PL before ensiling or PL containing two additives in terms of DMD and OMD were superior to the control silage. Ammonia nitrogen is an index for ruminal proteolysis. Some of the increase in ammonia nitrogen can be attributed to the higher content of CP in PL after ensiling.

Although high ammonia nitrogen concentration indicates high levels of ruminal degradable protein in the diet, lower concentrations of ammonia nitrogen may occur in similar conditions when the CP has a lower degradability coefficient or CP quality is low (Minson 1990). So, another part of increasing levels of ammonia nitrogen in PL after ensiling can be related to its higher ruminal degradable protein.

### Buffering capacity

Moharrery (2007) reported that the buffering system of ruminants is controlled by three essential mechanisms including, (1) the dietary additive buffers, (2) the buffering capacity of the feed consumed, and (3) the salivary buffer system (Moharrery 2007). The buffering capacity of some protein sources and leguminous fodders has been reported to be higher than 85 mEq × 10^–3^ (Montanez-Valdez et al. 2013), which is consistent with our study. In this study, the highest acid and also acid–base buffering capacity in PL ensiled 4% molasses indicated more acid is needed to change in pH of the water-soluble plant sample and high control of this plant in ruminal pH balance. Initial pH and titratable acidity have been reported to be the most critical determinants of rumen fluid pH. In the present study, the highest titratable acidity was observed for PL after ensiling (21.67 mEq × 10^–3^), indicating high resistance to acidification. By evaluating the pH and buffering capacity of the ration, we can predict the need for buffers to control and maintain rumen pH (Bujňák et al. 2011). All experimental silages had acidic pH and therefore, their consumption could lead to rumen pH reduction. It is reported that the amount and composition of minerals in the ash have a particular buffering effect on the plant's initial pH (Levic et al. 2005). Due to the different ash content of the present plant species (5.33–8.60%), their buffering capacity was also different.

In summary, ensiling of PL alone resulted in the loss of some nutrients, reduction of some minerals, reduction of gas production potential, and reduction of dry and organic matter digestibility, as well as reduction of TVFA production in the culture medium. Molasses and yogurt improved the fermentation characteristics of the silage environment, increased nutrient digestibility, and improved nutritional indicators and buffering parameters. In general, it is recommended that PL is ensiled with yogurt or molasses. Higher levels of yogurt or molasses (4%) than lower levels (2%) are recommended to add to the silo environment. In objective observations, we found that the silage containing 4% molasses had a better appearance quality and smell than other silages.

## Data Availability

The data will be made available upon reasonable request.

## Abbreviations

PL: Pomegranate leaves
CFU: Colony forming units
DM: Dry matter
EE: Ether extract
CP: Crude protein
NDF: Neutral detergent fiber
ADF: Acid detergent fiber
DMD: Dry matter digestibility
OMD: Organic matter digestibility
TVFA: Total volatile fatty acids
b_gas_: Potential gas production
c_gas_: Fractional rate of gas production
NFC: Non-fiber carbohydrates
ME: Metabolizable energy
NEl: Net energy for lactation

## References

[CR1] Aksu T, Baytok E, Bolat D (2004). Effects of a bacterial silage inoculant on corn silage fermentation and nutrient digestibility. Small Rumin Res.

[CR2] Ankom Technology. 2006a. Acid detergent fiber in feeds-filter bag technique. https://www.ankom.com/sites/default/files/document-files/Method_5_ADF_A200.pdf. Accessed 8 Jan 2021.

[CR3] Ankom Technology. 2006b. Neutral detergent fiber in feeds-filter bag technique. https://www.ankom.com/sites/default/files/document-files/Method_6_NDF_A200.pdf. Accessed 8 Jan 2021.

[CR4] AOAC (2005). Official methods of analysis.

[CR5] Babaeinasab Y, Rouzbehan Y, Fazaeli H, Rezaei J (2015). Chemical composition, silage fermentation characteristics, and in vitro ruminal fermentation parameters of potato-wheat straw silage treated with molasses and lactic acid bacteria and corn silage. J Anim Sci.

[CR55] Barnett AJG, Reid RL (1957). Studies on the production of volatile fatty acids from grass in artificial rumen. 1. Volatile fatty acids production from fresh grasses. J Agric Sci.

[CR6] Baytok E, Aksu T, Karsli MA, Muruz H (2005). The effects of formic acid, molasses and inoculant as silage additives on corn silage composition and ruminal fermentation characteristics in sheep. Turkish J Vet Anim Sci.

[CR7] Bujňák L, Maskaľová I, Vajda V (2011). Determination of buffering capacity of selected fermented feedstuffs and the effect of dietary acid-base status on ruminal fluid pH. Acta Vet Brno.

[CR8] Chabot DA, Chabot CD, Conway LK, Soto-Navarro SA (2008). Effect of fat supplementation and wheat pasture maturity on forage intake and digestion characteristics of steers grazing wheat pasture. J Anim Sci.

[CR9] Đorđević N, Koljajić V, Dinić B, Grubić G (2001). Conservation methods and effects of alfalfa use. Arch Agri Sci J.

[CR10] Ergün A, Tuncer ŞD, Çolpan İ, Yalçın S, Yıldız G, Küçükersan MK, Saçaklı P (2001). Hayvan besleme ve beslenme hastalıkları.

[CR11] Eyni B, Bashtani M (2016). Survey of nutritive value and degradability of sorghum silage from first and second cutting of forage. Res Anim Prod.

[CR12] Gandra JR, Oliveira ER, Takiya CS, Goes RHTB, Paiva PG, Oliveira KMP, Gandra ERS, Orbach ND, Haraki HMC (2016). Chitosan improves the chemical composition, microbiological quality, and aerobic stability of sugarcane silage. Anim Feed Sci Technol.

[CR13] Getachew G, Blümmel M, Makkar HPS, Becker K (1998). *In vitro* gas measuring techniques for assessment of nutritional quality of feeds: a review. Anim Feed Sci Technol.

[CR14] Getachew G, Robinson PH, DePeters EJ, Taylor SJ (2004). Relationships between chemical composition, dry matter degradation and in vitro gas production of several ruminant feeds. Anim Feed Sci Technol.

[CR15] He L, Zhou W, Wang Y, Wang C, Chen X, Zhang Q (2018). Effect of applying lactic acid bacteria and cellulase on the fermentation quality, nutritive value, tannins profile and in vitro digestibility of *Neolamarckia cadamba* leaves silage. J Anim Physiol Anim Nutr.

[CR16] Jasaitis DK, Wohlt JE, Evans JL (1987). Influence of feed ion content on buffering capacity of ruminant Feedstuffs in vitro. J Dairy Sci.

[CR17] Kazemi M (2019). Comparing mineral and chemical compounds, *in vitro* gas production and fermentation parameters of some range species in Torbat-e Jam. Iran J Rangel Sci.

[CR18] Kazemi M, Ghasemi Bezdi K (2021). An investigation of the nutritional value of camelthorn (*Alhagi maurorum*) at three growth stages and its substitution with part of the forage in Afshari ewes’ diets. Anim Feed Sci Technol.

[CR19] Kazemi M, Mokhtarpour A (2021). *In vitro* and *in vivo* evaluation of some tree leaves as forage sources in the diet of Baluchi male lambs. Small Rumin Res.

[CR20] Kazemi M, Tahmasbi AM, Naserian AA, Valizadeh R, Moheghi MM (2012). Potential nutritive value of some forage species used as ruminants feed in Iran. Afr J Biotechnol.

[CR21] Kazemi M, Valizadeh R (2019). Nutritive value of some rangeland plants compared to *Medicago sativa*. J Rangel Sci.

[CR22] Kazemi M, Valizadeh R (2021). The effect of dietary supplementation of ensiled pomegranate by-products on growth performance, nutrient digestibility, haematology parameters and meat characteristics of fat-tail lambs. Ital J Anim Sci.

[CR23] Kleinschmit DH, Kung L (2006). A meta-analysis of the effects of *Lactobacillus buchneri* on the fermentation and aerobic stability of corn and grass and small grain silages. J Dairy Sci.

[CR24] Knotek S (1997) Qualitative criteria of preserved forage and their effects on animal nutrition. In: Proceeding of the 8th International scientific symposium, Forage Conservation. pp: 37–49.

[CR25] Komolong MK, Barber DG, McNeill DM (2001). Post-ruminal protein supply and N retention of weaner sheep fed on a basal diet of lucerne hay (*Medicago sativa*) with increasing levels of quebracho tannins. Anim Feed Sci Technol.

[CR26] Krishnamoorthy U, Rymer C, Robinson PH (2005). The *in vitro* gas production technique: Limitations and opportunities. Anim Feed Sci Technol.

[CR27] Kung L, Shaver R (2001). Interpretation and use of silage fermentation analysis reports. Focus on Forage.

[CR28] Levic J, Prodanovic O, Sredanovic S (2005). Understanding the buffering capacity in feedstuffs. Biotechnol Anim Husb.

[CR29] Li M, Zi X, Zhou H, Hou G, Cai Y (2014). Effects of sucrose, glucose, molasses and cellulase on fermentation quality and *in vitro* gas production of king grass silage. Anim Feed Sci Technol.

[CR30] Li M, Zi X, Zhou H, Lv R, Tang J, Cai Y (2019). Silage fermentation and ruminal degradation of cassava foliage prepared with microbial additive. AMB Express.

[CR31] Makkar HP (2005). *In vitro* gas methods for evaluation of feeds containing phytochemicals. Anim Feed Sci Technol.

[CR32] Man NV, Wiktorsson H (2002). Effect of molasses on nutritional quality of cassava and gliricidia tops silage. Asian-Australas J Anim Sci.

[CR33] Mariotti M, Fratini F, Cerri D, Andreuccetti V, Giglio R, Angeletti FG, Turchi B (2020). Use of fresh scotta whey as an additive for alfalfa silage. Agronomy.

[CR34] Markham R (1942). A steam distillation apparatus suitable for micro-kjeldahl analysis. Biochem J.

[CR35] McDonald P (1981). The Biochemistry of Silage.

[CR36] McDonald P, Edwards RA, Greenhalgh JF, Morgan CA (2010). Animal nutrition.

[CR37] McDonald P, Henderson AR, Heron SJE (1991). The Biochemistry of Silage.

[CR38] Menke KH, Steingass H (1988). Estimation of the energetic feed value obtained from chemical analysis and *in vitro* gas production using rumen fluid. Anim Res Dev.

[CR39] Minson DJ (1990). Forages in ruminant nutrition.

[CR40] Moharrery A (2007). The determination of buffering capacity of some ruminant’s feedstuffs and their cumulative effects on TMR ration. Am J Anim Vet.

[CR41] Montanez-Valdez OD, Solano-Gama JDJ, Martinez-Tinajero JJ, Guerra-Medina CE, Coss ALD, Orozco-Hernandez R (2013). Buffering capacity of common feedstuffs used in ruminant diets. Rev Colomb Cienc Pecu.

[CR42] Muck RE (2010). Silage microbiology and its control through additives. R Bras Zootec.

[CR43] Nagaoka S, Kanauchi M (2019). Yogurt Production. Lactic acid bacteria methods in molecular biology.

[CR44] Natalello A, Luciano G, Morbidini L, Valenti B, Pauselli M, Frutos P, Biondi L, Rufino-Moya PJ, Lanza M, Priolo A (2019). Effect of feeding pomegranate by-product on fatty acid composition of ruminal digesta, liver, and muscle in lambs. J Agric Food Chem.

[CR45] Ni K, Wang F, Zhu B, Yang J, Zhou G, Pan Y, Tao Y, Zhong J (2017). Effects of lactic acid bacteria and molasses additives on the microbial community and fermentation quality of soybean silage. Bioresour Technol.

[CR46] Ørskov ER, McDonald I (1979). The estimation of protein degradability in the rumen from incubation measurements weighted according to rate of passage. J Agric Sci.

[CR47] Rabelo CHS, Härter CJ, Ávila CLDS, Reis RA (2019). Meta-analysis of the effects of *Lactobacillus plantarum* and *Lactobacillus buchneri* on fermentation, chemical composition and aerobic stability of sugarcane silage. Grassl Sci.

[CR48] Reyes-Gutiérrez J, Montañez-Valdez O, Guerra-Medina C, De Coss AL (2018). Effect of the inclusion of additives on the quality of sugarcane silage. Rev MVZ Cordoba.

[CR49] SAS (2002). Statistical analysis system. User’s guide: statistics.

[CR50] Sniffen CJ, O’Conno JD, Van Soest PJ, Fox DG, Russell JB (1992). A net carbohydrate and protein system for evaluating cattle diets. II. Carbohydrate and protein availability. J Anim Sci.

[CR51] Steg A, Van Der Meer JM (1985). Differences in chemical composition and digestibility of beet and cane molasses. Anim Feed Sci Technol.

[CR52] Taher-Maddah M, Maheri-Sis N, Salamatdoustnobar R, Ahmadzadeh A (2012). Estimating fermentation characteristics and nutritive value of ensiled and dried pomegranate seeds for ruminants using *in vitro* gas production technique. Open Vet J.

[CR53] Van Soest PV, Robertson JB, Lewis BA (1991). Methods for dietary fiber, neutral detergent fiber, and nonstarch polysaccharides in relation to animal nutrition. J Dairy Sci.

[CR54] Wang Y, He L, Xing Y, Zhou W, Pian R, Yang F, Chen X, Zhang Q (2019). Bacterial diversity and fermentation quality of Moringa oleifera leaves silage prepared with lactic acid bacteria inoculants and stored at different temperatures. Bioresour Technol.

